# Quality of life in caregivers of patients with schizophrenia: A literature review

**DOI:** 10.1186/1477-7525-7-84

**Published:** 2009-09-11

**Authors:** Alejandra Caqueo-Urízar, José Gutiérrez-Maldonado, Claudia Miranda-Castillo

**Affiliations:** 1 Department of Philosophy and Psychology, University of Tarapacá, 18 de Septiembre # 2222, Arica, Chile; 2 Department of Personality, Evaluation, and Psychological treatments, University of Barcelona, Paseo Valle de Hebron, 171, 08035 Barcelona, Spain; 3 Department of Mental Health Sciences, University College London, 67-73 Riding House Street, London, W1W 7EJ, UK

## Abstract

**Background:**

A couple of decades ago, hospitals or psychiatric institutions were in charge of caring for patients with schizophrenia; however, nowadays this role is performed by one or more patient's relatives. Evidence shows that informal caregivers experience negative changes in their quality of life (QOL). The aim of this study is to review the main factors associated with the QOL of caregivers of people with schizophrenia.

**Methods:**

A search through databases from journals published last decade between 1998 and 2008 was performed. In accordance with the inclusion criteria, titles and abstracts of citations obtained from the search were examined independently by two authors and irrelevant articles discarded. The full text of those studies considered relevant by either reviewer were obtained and assessed independently. Where differences of opinion rose they were resolved by discussion. Out of the 258 references, 37 were included in the review.

Studies which assessed factors associated with caregivers of people with schizophrenia's quality of life were included and the information summarized.

**Results:**

Evidence suggest that physical, emotional and economic distress affect negatively caregiver's QOL as a result of a number of unfulfilled needs such as, restoration of patient functioning in family and social roles, economic burden, lack of spare time, among other factors.

**Conclusion:**

Decreased QOL may be associated with caregivers' burden, lack of social support, course of the disease and family relationships problems. In addition, in developing countries, QOL is affected by caregivers' economic burden. High quality research is needed in order to identify factors associated with QOL over time and testing the efficacy of interventions aiming to improve QOL in caregivers of patients with schizophrenia.

## Review

### Caregiver quality of life

Nowadays family has taken functions which were performed in the past by psychiatric institutions. This change highlights not only the relevance of emotions and affections within a family, but also the great amount of burden experienced by these relatives while taking care of a psychotic patient as well. Thus, "informal care" is playing a significant role in development and evaluation of health programs and policies [1].

Main caregiver has been defined as the person belonging to the patient's informal support system who takes the care and is responsible for the patient, and who commits most of his or her time to that task without receiving any economic retribution [2]. There is plenty of research about burden on relatives of psychotic patients; however concern for this group has increased during last decades [3-5]. Dillehay and Sandys [6] defined family burden as a "psychological state produced by the combination of physical work, emotional pressure, social restrictions, and economic demands arising from taking care for a patient as well". Burden on relatives of patients with schizophrenia has been found associated with an important reduction in their QOL, causing damage in caregiver's health condition [7-9].

In 1993, The WHOQOL (The World Health Organization Quality of Life) Group [10] defined QOL as individuals' perceptions of their position in life in the context of the culture and value systems in which they live and in relation to their goals, expectations, standards and concerns. It is a wide concept implying many aspects and many interpretations have come from it. QOL concept comprises different dimensions: individual's physical and emotional health, psychological and social well-being, fulfillment of personal expectations and goals, economic assurance, and finally functional capacity to develop daily routines normally [11,12].

Martens y Addington [13] observed that family members are significantly stressed as a result of having one of them with schizophrenia. Ivarsson, Sidenvall, and Carlsson [14] agreed that burden on a family caregiver is complex and includes many areas, such as daily life, worries, and social pressure. In addition, empirical evidence confirms that caring for a patient with schizophrenia generates economic burden on the family. Gutiérrez-Maldonado and colleagues [15] carried out a study with a Chilean sample confirming that, disregarding the level of development of a country; caregivers experience high levels of burden.

In the health field, QOL (a construct closely related to burden) is one of the most important components associated with delivering an integral service to an ill person and their family, emphasizing the subjective perspective held by the patient and the family. In this context, one of the main objectives is the development of supporting activities rather than reducing symptoms and preventing relapses only.

Until recently, due to the inclusion of new drugs, research on QOL has been focused on schizophrenia patients rather than their caregivers. Currently, informal and formal caregivers are being considered as a valuable component of an integral treatment for patients, thus their QOL and burden are being evaluated [16-18].

This review tries to answer the following questions: a) What variables are related to QOL damage in caregivers of patients with schizophrenia? b) Does relative's QOL differ substantially among developed and developing countries? c) Which objectives should aim at future researches.

## Methods

### Search Strategy

A systematic search was carried out using the following electronic databases: MEDLINE via PubMed, Web of Science and PsycINFO. The following keywords were used: quality of life, burden, schizophrenia, families, caregivers, and a combination of them. The search comprised primary and secondary studies and was limited to references published from 1998 to 2008. Potential studies were included if they considered a primary family caregiver and focused on caregiver's QOL related to schizophrenia. Since this review aimed to identify variables related to QOL in caregivers of patients with schizophrenia, descriptive, cross-sectional and qualitative studies were included.

The hits retrieved by database were: MEDLINE via Pubmed (73), Web of Science (93) and PsycINFO (49). In accordance with the inclusion criteria, titles and abstracts of citations obtained from the search were examined independently by two authors and irrelevant articles discarded. The full text of those studies considered relevant by either reviewer were obtained and assessed independently. Where differences of opinion rose they were resolved by discussion. Out of the 258 references, 37 were included in the review.

## Results

### Methodological aspects

Most reviewed studies had a "cross-sectional" design showing a low level of evidence available. The less were of "prospective cohort" type, only one of them was a "case control" study, and a study alone used qualitative methodology. To assess caregiver QOL, different methods and instruments were used; therefore, comparison among studies was difficult. The amount of caregivers interviewed ranged from 30 to 288. It is likely that some studies were not sensitive enough to detect any significant association with caregiver burden and/or caregiver QOL.

The correlations between different variables and caregiver's QOL included in the discussion came mainly from descriptive studies. In order to summarize the results taking into account the heterogeneity and the poor quality of the studies, the authors agreed that an association between any factor and caregiver's QOL was considered in the discussion when it was found statistically significant in the original paper (p < 0.05) and was present in more than 10% of the papers reviewed. This allowed us to discuss those associations that were significant form a statistical point of view and more frequently studied.

### Factors associated with caregiver QOL

Additional file 1 displays associated variables found in studies on QOL of relatives of patients with schizophrenia.

Despite differences among countries, studies carried out in different parts of the world show similar outcomes. Following we describe the factors that were found associated with caregivers' QOL.

Several studies stated that appearance of psychotic symptoms or the course of the disease produce an important level of burden [19-37].

Caregivers' health was highly deteriorated. Stress problems, anxiety, and depression were observed in several studies [20-34]. Interestingly, Dyck et al. [35] found that caregivers catch infectious diseases. This could have occurred due to a deterioration in the caregiver's immune system.

Working life was also significantly affected. Caregivers must leave their jobs, modify their working hours or change to another job. Moreover, in some cases, stress seemed to be associated with a triple shift: job, household duties, and care for a patient [36-39].

The last dimension evaluated was economic burden. Economy issues produce concern in caregivers due to expenses in different areas, e.g. drug therapy and treatment. Regarding this, there is a difference between developed and developing countries. In countries such as Chile, Nigeria, and India caregivers expressed more concern in this dimension, likely caused by scarcity of community and health resources [5,9,21,22,24,40].

Family dynamics were affected due to the presence of disagreements, conflicts, and even violence among its members. In addition, some close relatives might go away avoiding having to take care of the patient [5,16,21,37,39-42].

Damage in caregiver social life and a lack of social support has lead to caregivers complaints towards treatment delivered by health institutions and their professionals [5,10,19,21,22,32,34,37,38,40,43,44]. Jungbauer et al. [22] found that sometimes professionals disqualify informal caregivers.

Some researches have analyzed cultural variables in populations that have not been frequently included in research. Caregivers belonging to an ethnic minority have the worst QOL and those from countries with more traditional families use religion as a way of coping [29,32,34,43,45].

Finally, most of the studies show that the mother is the one who takes main care of the patient and has worse QOL than other type of informal caregivers caused likely by her caring chores [5,15,19,22,30,35,38].

## Conclusion

The variety of methods used in the reviewed studies makes comparison among them difficult. It would be important to achieve an agreement regarding what instrument is the most appropriate to employ in the case of caregivers of patients with schizophrenia in order to measure their QOL. In addition, future research is needed going beyond the cross-sectional design. It should be considered at least a case-control design having comparative groups clearly defined and blind (for QOL evaluator), or studies using self-report instruments to measure this construct and other variables in which disturbance variables are in control. This would allow obtaining better evidence about factors associated with QOL of caregivers of patients with schizophrenia.

Despite the fact that good quality evidence is required, a recent trend towards studying this subject on diverse family groups can be observed.

Regarding the aims of this review, some similarities were found in the results obtained from the studies reviewed:

a) What variables are related to QOL damage in caregivers of patients with schizophrenia?

Main variable was emotional burden on caregivers as a consequence of their role, lack of social and working support, course of the disease, and disruptions in family life. All these factors were associated with a considerable damage of QOL.

b) Does relatives' QOL differ substantially among developed and developing countries?

The feeling of being exhausted seems to be generalized to relatives from different countries as well as cost associated with mental disorders, however, major differences regarding QOL appear to be related to having a better access to and higher availability of health and economic resources for these caregivers. In developing countries, economic burden may be playing an important role in relative's QOL. Lack of psychiatrist, day hospitals, access to drug treatments, among others, could generate a considerable concern in these relatives.

c) Which objectives should aim at future researches?

There is a need for treatment and follow-up as much as improvements in family intervention programs delivered by health services. These factors should be considered in future research in this area. Program design should take into account socio-cultural characteristics of the population attending a health service. Efficacy of such interventions needs to be proved since they could be beneficial not only for the patients, but also for the caregivers.

This review has limitations. The study design of most reports was cross-sectional reflecting the lowest level of evidence. This has the following implications for the results:

a) Associations found across studies may have been influenced by different sources of bias making the internal and external validity of them questionable. For example, recruitment strategies and sampling varied a lot across studies. Most of the studies employed purposive samples where interviews were applied only to those who were willing to participate in a research. Therefore, caregivers studied may have not been a representative sample of caregivers of patients with schizophrenia.

b) The criteria adopted for including associations in the discussion might have excluded those that maybe were clinically significant but did not reach statistical significance.

c) The descriptive nature of most of the included studies made the use of quantitative pooling methods (e.g. meta-analysis) not possible. As mentioned previously, good quality studies are needed in order to assess the impact of interventions aimed to improve caregivers of patients with schizophrenia's QOL.

In summary, it seems that "informal care" is playing an important role in the care of patients with schizophrenia and this issue needs to be thoroughly analyzed because of the high psychopathological risk experienced by informal carergivers.

## Abbreviations

QOL: Quality of life

## Competing interests

The authors declare that they have no competing interests.

## Authors' contributions

ACU contributed to the design and coordination of the study. JGM was responsible for primary study design and supervision of data collection. CMC was a methodologic consultant, assisted with data analysis and interpretation, and participated in manuscript editing. All authors read and approved the final manuscript.

## Supplementary Material

Additional file 1 **Variables associated with QOL in family caregivers of patients with schizophrenia**. The table shows the variables associated with QOL in family caregivers of patients with schizophrenia found in the literature review [46-53]. Click here for file
