# Supplementary material for: Quality of life in caregivers of patients with schizophrenia: A literature review
Source: Health Qual Life Outcomes. 2009 Sep 11;7:84. doi: 10.1186/1477-7525-7-84 (PMC2749816; doi:10.1186/1477-7525-7-84)
Supplement: Additional file 1 — Variables associated with QOL in family caregivers of patients with schizophrenia. The table shows the variables associated with QOL in family caregivers of patients with schizophrenia found in the literature review [46-53]. [file 1477-7525-7-84-S1.doc]

## Additional file 1 - Variables associated with QOL in family caregivers of patients with schizophrenia

| Authors | Coping | Satisfaction and relationship with Health services | Social stigmatization | Ethnics and cultural aspects | Emotional burden | Economic burden | Family relationship | Relative’s working life | Relative’s social life | Social support | Relative’s psychopathological risk | Family intervention | Relationship | Social functioning and disease course |
| --- | --- | --- | --- | --- | --- | --- | --- | --- | --- | --- | --- | --- | --- | --- |
| [41] |  |  |  |  |  |  |  |  |  |  |  |  |  |  |
| [53] |  |  |  |  |  |  |  |  |  |  |  |  |  |  |
| [19] |  |  |  |  |  |  |  |  |  |  |  |  |  |  |
| **[35]** |  |  |  |  |  |  |  |  |  |  |  |  |  |  |
| [21] |  |  |  |  |  |  |  |  |  |  |  |  |  |  |
| [38] |  |  |  |  |  |  |  |  |  |  |  |  |  |  |
| [51] |  |  |  |  |  |  |  |  |  |  |  |  |  |  |
| [23] |  |  |  |  |  |  |  |  |  |  |  |  |  |  |
| [22] |  |  |  |  |  |  |  |  |  |  |  |  |  |  |
| [37] |  |  |  |  |  |  |  |  |  |  |  |  |  |  |
| [52] |  |  |  |  |  |  |  |  |  |  |  |  |  |  |
| [39] |  |  |  |  |  |  |  |  |  |  |  |  |  |  |
| **[36]** |  |  |  |  |  |  |  |  |  |  |  |  |  |  |
| [50] |  |  |  |  |  |  |  |  |  |  |  |  |  |  |
| [40] |  |  |  |  |  |  |  |  |  |  |  |  |  |  |
| [9] |  |  |  |  |  |  |  |  |  |  |  |  |  |  |
| [46] |  |  |  |  |  |  |  |  |  |  |  |  |  |  |
| [24] |  |  |  |  |  |  |  |  |  |  |  |  |  |  |
| [25] |  |  |  |  |  |  |  |  |  |  |  |  |  |  |
| [42] |  |  |  |  |  |  |  |  |  |  |  |  |  |  |
| [15] |  |  |  |  |  |  |  |  |  |  |  |  |  |  |
| [30] |  |  |  |  |  |  |  |  |  |  |  |  |  |  |
| [31] |  |  |  |  |  |  |  |  |  |  |  |  |  |  |
| [45] |  |  |  |  |  |  |  |  |  |  |  |  |  |  |
| [47] |  |  |  |  |  |  |  |  |  |  |  |  |  |  |
| [32] |  |  |  |  |  |  |  |  |  |  |  |  |  |  |
| [26] |  |  |  |  |  |  |  |  |  |  |  |  |  |  |
| [27] |  |  |  |  |  |  |  |  |  |  |  |  |  |  |
| [48] |  |  |  |  |  |  |  |  |  |  |  |  |  |  |
| [33] |  |  |  |  |  |  |  |  |  |  |  |  |  |  |
| [34] |  |  |  |  |  |  |  |  |  |  |  |  |  |  |
| [28] |  |  |  |  |  |  |  |  |  |  |  |  |  |  |
| [49] |  |  |  |  |  |  |  |  |  |  |  |  |  |  |
| [29] |  |  |  |  |  |  |  |  |  |  |  |  |  |  |
